# Behind the Mask: Risk Profiles of Depression and Anxiety in Anesthesiology Residents

**DOI:** 10.1155/da/9253231

**Published:** 2026-07-16

**Authors:** Diana Guízar-Sánchez, Ana Fresán, Ricardo-Arturo Saracco-Alvarez, María Yoldi-Negrete, Rebeca Robles-García, Carlos-Alfonso Tovilla-Zárate

**Affiliations:** ^1^ Department of Physiology, Faculty of Medicine, National Autonomous University of Mexico (UNAM), Escolar 411A, Copilco Universidad, Coyoacán, Mexico City, 04360, Mexico, unam.mx; ^2^ Biomedical Research in Mental Health Directorate, National Institute of Psychiatry, Ramón de la Fuente Muñiz, Calz. México-Xochimilco, 101, Mexico City, 14370, Mexico, inprf.gob.mx; ^3^ Center for Global Mental Health Research, National Institute of Psychiatry, Ramón de la Fuente Muñiz, Calz. México-Xochimilco, 101, Mexico City, 14370, Mexico, inprf.gob.mx; ^4^ Multidisciplinary Academic Division of Comalcalco, Juárez Autonomous University of Tabasco, Ranchería Sur Cuarta Sección, Comalcalco, 86205, Tabasco, Mexico, ujat.mx

**Keywords:** anesthesiology residents, anxiety, depression, doctor’s healthcare, mental health

## Abstract

**Introduction:**

Anesthesiology residents play a crucial role in healthcare, yet the demanding nature of their residency—long hours, high stress, and challenging work conditions—often leads to mental health issues like depression and anxiety. Despite the importance of resident well‐being, mental health problems among anesthesiology residents in Mexico remain underexplored. The main objective of this study was to characterize potential profiles linked to anxiety, depression, and their comorbidity among anesthesiology residents, drawing on demographic and residency‐related variables.

**Materials and Methods:**

A cross‐sectional study was conducted with 1208 anesthesiology residents who completed an online survey between October 2019 and April 2021. The survey collected demographic data, professional activities, adversities, and mental health, using the ICD‐11 PHC screening test.

**Results:**

A total of 74.5% of residents suffered from no symptoms of depression or anxiety (C), 5.3% suffered from depression (D), 13.1% suffered from anxiety (A), and 7.1% suffered from both (AD). Sleep deprivation (average of 5.1 h per night) was common, with residents reporting more sleep disturbances and work‐related distress, particularly those suffering from depression or comorbid depression and anxiety. Logistic regression showed that depression was associated with being single and sleeping fewer hours, while residents with anxiety or both conditions reported higher work‐related distress and greater exposure to assaults.

**Discussion:**

This study emphasized the need for systemic changes aimed at a more supportive training environment, including interventions to protect residents’ sleep, which will enhance well‐being and provide mental health care to those who need it.

## 1. Introduction

Medical specialization is a vital, ongoing education process for physicians, which is essential for meeting the population’s healthcare needs. In Mexico, medical residents are crucial not only for future training but also as a key part of the healthcare workforce [1]. In addition to their traditional role in the intensive care of patients during surgery and other medical procedures, anesthesiologists conduct preoperative evaluations, provide postoperative care, lead intensive care unit teams, deliver care in nonoperating room settings, and practice pain medicine [2].

Anesthesiologists are expected to seek the well‐being of their patients, guaranteeing their protection from physical, emotional, or social harm while ensuring excellence in terms of the quality of care provided [3]. This is merely an ideal that does not reflect the everyday clinical practice for anesthesiology residents. Complex cases, unforeseen medical complications, coping with patients’ suffering, life‐threatening circumstances, and death make it impossible to provide an absolute guarantee for any medical procedure.

Anesthesia training can represent an intensely challenging experience. The physical separation of the anesthesiologist from the surgical team—marked by the sterile drape that separates them [4]—combined with long hours, high job demands, unpredictable schedules, frequent night shifts, sleep deprivation, a difficult work environment, and poor work–life balance may contribute to a deeply isolating experience [5], resulting in a sense of disconnection and “thwarted belonging.” Additionally, residents often face hostile and stigmatizing environments, where interactions with senior peers, colleagues from different medical specialties, patients, and patients’ families can lead to conflict [6, 7]. Furthermore, residents frequently feel unsupported due to cultural norms and societal demands (residents navigate the tension between growing clinical responsibility and limited decision‐making authority), which exacerbates their emotional and psychological stress [8]. These challenges contribute to increased stress and mental health problems, such as depression and anxiety, making anesthesiology residents a particularly vulnerable group [9, 10].

In a national survey conducted in the United States, a questionnaire was sent to 1000 randomly selected anesthesiology residents. Of the 384 residents who responded, 15% screened positive for depression. The survey found that longer working hours were associated with greater probability of depression, while being married or in a domestic partnership was linked to less likelihood of depression [11]. In a cross‐sectional survey conducted in an intensive care unit in Turkey, more than half of the residents (54.3%, *n* = 35) exhibited moderate to severe anxiety symptoms, compared to just 21% (*n* = 26) of the attending physicians surveyed [12]. These findings highlight how anxiety may be more prevalent among residents, probably because of their greater exposure to clinical procedures and due to the academic pressures they face alongside their clinical duties during residency, an issue that warrants further study, particularly among representative samples of anesthesiology residents.

Given the unique demands of their work, it appears inevitable that anesthesiology residents are likely to experience depression, anxiety, or both. However, the extent of mental health problems among anesthesiology residents in Mexico is currently unknown. Therefore, the main objective of this study was to characterize potential profiles linked to anxiety, depression, and their comorbidity among anesthesiology residents, drawing on demographic and residency‐related variables.

## 2. Materials and Methods

### 2.1. Participants

This cross‐sectional, comparative, and retrospective study centers on anesthesiology residents in Mexico who completed an online mental health survey. It analyzes data from this specific group within a larger cohort of medical specialty residents originally collected between 2019 and 2021 from 31 of the 32 federal states in Mexico as part of a broader research project [6].

To become an anesthesiology resident in Mexico, candidates who have graduated from an accredited medical school must pass the National Examination for Medical Residency Applicants (ENARM), administered by the Interinstitutional Commission for the Training of Human Resources for Health (CIFRHS) [13]. If candidates pass the exam, they are selected for residency positions in healthcare institutions that offer specialization in anesthesiology. According to the records of the Comprehensive System of the UNAM Postgraduate Studies Division and the CIFRHS, there were 1363 registered anesthesiology residents, of whom 1208 responded to the survey, indicating a response rate of 88.6%.

### 2.2. Assessment Procedure

The survey, conducted in Spanish, took ~25–30 min to complete and consisted of four sections:

1. Demographic information: this section collected data on participants’ age at the time of the study, as well as sex (man/woman) and marital status (single/partnered).
2. Professional activities: current year of residency (first to fourth year of residency), maximum number of hours worked in a single day (excluding continuous 36‐h shifts related to on‐call duties), assessing hours of sleep, and whether participants considered their sleep to be restful (subjective perception of obtaining sufficient and refreshing sleep that allows the individual to feel physically and mentally recovered upon awakening) or restless (subjective perception of disturbed or nonrestorative sleep characterized by frequent awakenings, discomfort, or difficulty achieving continuous and refreshing sleep) were assessed.
3. Self‐reported professional adversities: participants were asked to reflect on their experiences from the beginning of their medical residency up until the time of evaluation. Assaults were assessed with the question, “Have you ever experienced physical, verbal, or psychological assaults since starting your medical residency?” (answer: yes/no). Those who responded in the affirmative were subsequently asked, “Who were the primary perpetrators of these assaults?” Respondents could specify whether the assaults came from patients (yes/no response option), patients’ relatives (yes/no response option), or senior colleagues (yes/no response option). Additionally, participants were asked whether they had been directly involved in the care of patients suffering from suicidal ideation, patients who had later committed suicide, or patients who had died under their care. All these questions had a yes/no response option.
4. Mental health: the ICD‐11 PHC screening test was used, which presents five items on the anxiety scale and five items on the depression scale. A score of 3 or higher on each scale has a positive predictive value of 84% for identifying individuals with a current clinical diagnosis of depression and 89% for those among the Mexican population with a clinical diagnosis of anxiety [14]. Based on these cut‐off points, residents were categorized into (1) those with no symptoms of anxiety or depression, (2) those suffering from depression, (3) those suffering from anxiety, and (4) those suffering from both anxiety and depression. Perceived work‐related distress was assessed with the following question: “What level of distress do you perceive due to your profession?” Participants’ response was registered on a visual analog scale, represented by a 100 mm horizontal line with word descriptors at each end (0 = no distress at all; 100 = maximum perceived distress) [15].

To distribute the survey, researchers initially invited residents known to them via email; this included a link to the survey. The residents were encouraged to share the email with colleagues nationwide. This chain‐referral method was used to maximize outreach within the resident community. Additionally, the survey link was made available at the conclusion of the annual residency exam at the Faculty of Medicine, Universidad Nacional Autónoma de México (UNAM). The survey link was restricted and managed by a nonprofit organization, dedicated to disseminating mental health information.

### 2.3. Ethical Considerations

The survey content and all study procedures were approved by the Ethics and Research Committees of the Ramón de la Fuente Muñiz National Institute of Psychiatry, Secretariat of Health, Mexico (Approval Numbers CEI/ C/018/2019 and IC19119.0). Duplicate entries were identified through a combination of the IP address and date of birth, with the most recent submission retained. Participants took part in this study on a voluntary basis, were not paid for their participation, and could withdraw their consent by not completing the online survey. Anonymity was ensured, and participants had the option to withdraw at any time by exiting the survey; therefore, only complete surveys were saved to the database. In this way, any questionnaire that was not completed in full was removed from the database and excluded from the analyses as noncompletion was considered indicative of the participant’s withdrawal of consent to continue in the study.

### 2.4. Statistical Analysis

Descriptive statistics were first computed, including frequencies and percentages for categorical variables and means and standard deviations (S.D.) for continuous variables. The four sections of the survey were compared between the control participants (C—suffering from no anxiety or depression), participants suffering from depression (D), participants suffering from anxiety (A), and those suffering from both anxiety and depression (AD) using chi‐square tests (*χ* ^2^) and analysis of variance (ANOVA) with Bonferroni correction, where appropriate. Skewness and kurtosis values for the continuous variables (age, maximum working hours per day, hours of sleep, and work‐related distress) were within acceptable ranges. Skewness values ranged from −0.36 to 0.87, and kurtosis ranged from −1.39 to 1.44, indicating no substantial deviations from normality. In addition, Levene’s test for these variables was nonsignificant (*p*  > 0.05), supporting the assumption of homogeneity of variances. The effect size for comparisons with significant differences was determined with Cramer’s *V* for the comparison of categorical variables and with the eta‐squared (*ƞ* ^2^) with reference values for the effect size estimation interpretation as 0.01 = small, 0.06 = medium, and 0.14 = large [16].

Following comparative analysis, a multinomial logistic regression was performed, which included the variables where significant differences arise between groups as independent variables. The classification into groups based on the presence or absence of depression and anxiety served as the dependent variable with the C group as the reference. Considering at least 10 predictor variables and 50 cases per predictor, a sample size of 600 subjects was required for a nonbinary dependent variable with four possible outcomes (C, A, D, and AD) for the model [17]. To determine the model’s goodness of fit, the Pearson chi‐square test was used, and the Nagelkerke *R* ^2^ was determined to identify the variance explained by the model. Regression coefficients (*β*), S.D. of *β*, odds ratios (OR), and 95% confidence intervals are reported.

All analyses were conducted using data from participants who completed the survey in full; therefore, no missing data were present, and no data imputation methods were required. The alpha value for tests was set at *p* ≤ 0.05 and all analyses were performed using SPSS version 26 for Windows, PC.

## 3. Results

From the 1208 residents who participated in the online survey, 74.5% (*n* = 900) were placed in the C group (not suffering from depression or anxiety), 5.3% (*n* = 64) met the cut‐off score for depression (D group), 13.1% (*n* = 158) for anxiety (A group), and the remaining 7.1% (*n* = 86) met the cut‐off point for both depression and anxiety (AD group), based on the ICD‐11 PHC screening test.

### 3.1. Demographic Features

The mean age of the participants was 29.3 (S.D. = 2.7) years. A slightly higher proportion of participants were women (60.4%, *n* = 730) and single (53.6%, *n* = 647). A higher percentage of participants from the AD group were single (54.7%, *n* = 278) when compared to the other groups (Bonferroni < 0.05), while no differences were found in terms of age and sex (see Table 1).

**Table 1:** Demographics, professional activities and adversities, affecting mental health among anesthesiology residents with and without depression and/or anxiety.

Variables	Control (C) *n* = 900	Depression (D) *n* = 64	Anxiety (A) *n* = 158	Anxiety/ depression (AD) *n* = 86	Statistic
Demographic features					

Sex *n* (%)					
Men	363 (40.3)	23 (35.9)	61 (38.6)	31 (36.0)	*χ* ^2^ = 1.0, df 3, *p* = 0.78
Women	537 (59.7)	41 (64.1)	97 (61.4)	55 (64.0)	
Age—years *mean (S.D.)*	29.2 (2.7)	29.3 (2.9)	29.5 (2.8)	29.2 (2.9)	*F* = 0.6, df 3, *p* = 0.60
Marital status *n* (%)					
Single	488 (54.2)	26 (40.6)	77 (48.7)	56 (65.1)	*χ* ^2^ = 10.5, df 3, *p* = 0.01 Cramer’s *V* = 0.09
Partnered	412 (45.8)	38 (59.4)	81 (51.3)	30 (34.9)	

Professional activities					

Year of residency *n* (%)					
First	150 (16.7)	12 (18.8)	24 (15.2)	18 (20.9)	*χ* ^2^ = 11.3, df 9, *p* = 0.25
Second	323 (35.9)	33 (51.6)	55 (34.8)	28 (32.6)	
Third	350 (38.9)	15 (23.4)	61 (38.6)	32 (37.2)	
Fourth	77 (8.6)	4 (6.3)	18 (11.4)	8 (9.3)	
Maximum working hours per day *mean (S.D.)*	19.0 (4.9)	18.7 (5.0)	19.4 (4.9)	20.1 (5.0)	*F* = 1.4, df 3, *p* = 0.22
Restful sleep—yes *n* (%)	363 (40.3)	13 (20.3)	55 (34.8)	23 (26.7)	*χ* ^2^ = 15.8, df 3, *p* = 0.001 Cramer’s *V* = 0.11
Hours of sleep *mean (S.D.)*	5.2 (1.1)	4.7 (0.8)	5.1 (0.9)	5.0 (1.1)	*F* = 3.5, df 3, *p* = 0.01 *ƞ* ^2^ = 0.009

Self‐reported professional adversities *n* (%)					

Physical assault—yes	253 (28.1)	23 (35.9)	61 (38.6)	38 (44.2)	*χ* ^2^ = 15.4, df 3, *p* = 0.001 Cramer’s *V* = 0.11
Verbal assault—yes	227 (25.2)	19 (29.7)	50 (31.6)	41 (47.7)	*χ* ^2^ = 21.1, df 3, *p* < 0.001 Cramer’s *V* = 0.13
Psychological assault—yes	247 (27.4)	23 (35.9)	60 (38.0)	40 (46.5)	*χ* ^2^ = 19.3, df 3, *p* < 0.001 Cramer’s *V* = 0.12
Assault by patients—yes	162 (18.0)	15 (23.4)	46 (29.1)	32 (37.2)	*χ* ^2^ = 24.8, df 3, *p* < 0.001 Cramer’s *V* = 0.14
Assault by patients’ relatives—yes	142 (15.8)	8 (12.5)	36 (22.8)	25 (29.1)	*χ* ^2^ = 14.0, df 3, *p* = 0.003 Cramer’s *V* = 0.10
Assault by colleagues—yes	118 (13.1)	14 (21.9)	35 (22.2)	30 (34.9)	*χ* ^2^ = 33.8, df 3, *p* < 0.001 Cramer’s *V* = 0.16
Care of patients with suicide ideation—yes	229 (25.4)	18 (28.1)	57 (36.1)	22 (25.6)	*χ* ^2^ = 7.8, df 3, *p* = 0.04 Cramer’s *V* = 0.08
Care of patients who committed suicide—yes	71 (7.9)	7 (10.9)	17 (10.8)	8 (9.3)	*χ* ^2^ = 2.0, df 3, *p* = 0.56
Care of patients who died—yes	355 (39.4)	31 (48.4)	76 (48.1)	30 (34.9)	*χ* ^2^ = 6.9, df 3, *p* = 0.07

Mental health *mean (S.D.)*					

Work‐related distress	50.1 (29.4)	52.9 (31.9)	63.9 (25.0)	69.4 (23.5)	*F* = 20.0, df 3, *p* < 0.001 *ƞ* ^2^ = 0.04

### 3.2. Professional Activities and Mental Health

A higher proportion of anesthesiology residents were in the third (37.9%, *n* = 458) and second (36.3%, *n* = 439) years of their residency, 16.9% (*n* = 204) were in the first year, and the remaining 8.9% (*n* = 107) were in the fourth year of residency. Excluding the 36‐h continuous on‐call shift, the mean maximum number of hours spent on professional activities per day was 19.7 h (S.D. = 4.9). The average number of hours of sleep reported by the residents was 5.1 h (S.D. = 1.1). Perceived work‐related distress was moderate according to the mean score of 53.4 (S.D. = 29.3) registered on the visual analog scale.

As shown in Table 1, no differences were observed between groups in terms of year of residency or concerning the maximum number of hours dedicated to professional activities. The residents in group D reported fewer hours of sleep compared to those in group C (Bonferroni < 0.01). Both group D and group AD showed a lower percentage of residents with restful sleep compared to the C and A groups (Bonferroni < 0.01). Residents in groups A and AD reported higher levels of work‐related distress (Bonferroni < 0.001).

### 3.3. Self‐Reported Professional Adversities

Physical assaults were reported by 31% (*n* = 375) of anesthesiology residents, followed by 30.6% (*n* = 370) who reported psychological assaults and 27.9% (*n* = 337) who reported verbal assaults. Being assaulted was more frequent among residents from the AD group (Bonferroni < 0.01). Similar percentages were observed in the A and D groups, while the C group reported the fewest incidents of assault. The primary perpetrators of assaults identified by residents were patients (21.1%, *n* = 255), followed by patients’ relatives (17.5%, *n* = 211) and senior colleagues (16.3%, *n* = 197). Residents in the AD group reported higher frequencies of assaults from all three perpetrators (Bonferroni < 0.01), while the lowest frequencies were reported by the C group, except for assaults by patients’ relatives, which were slightly less frequent among residents in the D group. These findings are presented in Table 1.

Less than 10% of participants had been directly involved in the care of patients who later committed suicide, while 27% (*n* = 326) had been involved in the care of patients with suicide ideation and 40.7% (*n* = 492) had been involved in the care of patients who later died. The four groups of anesthesiology residents reported similar frequencies for these variables, except for residents in group A (Bonferroni < 0.05), who reported a higher incidence of having patients with suicidal ideation under their care (Table 1).

### 3.4. Variables Associated With Anxiety, Depression, or Both Among Anesthesiology Residents

The following variables were included in the multinomial logistic regression model, manifesting differences between groups: (1) marital status, (2) hours of sleep, (3) restful sleep, (4) work‐related distress, (5) physical assault, (6) verbal assault, (7) psychological assault, (8) assault perpetrated by patients, (9) assault perpetrated by patients’ relatives, (10) assault perpetrated by senior colleagues, and (11) involved in the care of patients with suicidal ideation. A multicollinearity test was performed on the six assault variables included in the model. Collinearity tolerance and variance inflation factor (VIF) were in adequate ranges (tolerance 0.22–0.56 and VIF 1.7–4.5) with the exception of physical and psychological assault (tolerance 0.05 and 0.04 and VIF 18.3 and 20.5, respectively).

Results from the multinomial logistic regression in this cross‐sectional study are displayed in Table 2 and indicate associations between resident groups and several outcomes. The model displayed marginal goodness of fit (Pearson *χ* ^2^ goodness‐of‐fit = 1534.36, *p* = 0.05) and the contribution of the variables to the model, according to the Nagelkerke *R* ^2^ was 13.3%. The model indicates that residents from group D are more likely to be single (OR = 1.75) and report fewer hours of sleep (OR = 0.76) than residents from group C. Restful sleep was less frequently reported by residents from groups D and AD (OR = 0.47 and OR = 0.58). Residents in group A were more likely to be involved in the care of patients with suicidal ideation (OR = 1.42), and work‐related distress was higher in A (OR = 1.01) and AD residents (OR = 1.02). Exposure to assaults from patients (OR = 2.19) and senior colleagues (OR = 2.71) was associated with higher odds of anxiety and depression among anesthesiology residents.

**Table 2:** Multinomial logistic regression analysis including demographics, professional activities and adversities that affect mental health and identify group to which anesthesiology residents belong.

Variables	(*β*)	(*β*) S.D.	OR	95% C.I.	(*β*)	(*β*) S.D.	OR	95% C.I.	(*β*)	(*β*) S.D.	OR	95% C.I.
	Depression vs. control (D vs. C)				Anxiety vs. control (A vs. C)				Anxiety/depression vs. control (AD vs. C)			
Marital status—single	0.56 ^∗^	0.26	1.75	1.03–2.95	0.20	0.17	1.22	0.86–1.72	−0.40	0.24	0.66	0.40–1.08
Restful sleep—yes	−0.74 ^∗^	0.33	0.47	0.24–0.92	−0.19	0.19	0.82	0.55–1.21	−0.54 ^∗^	0.28	0.58	0.33–0.99
Hours of sleep	−0.27 ^∗^	0.12	0.76	0.59–0.97	0.007	0.08	1.008	0.85–1.19	0.002	0.11	1.002	0.79–1.26
Physical assault—yes	−0.73	1.29	0.48	0.03–6.11	−0.39	0.77	0.67	0.14–3.05	−1.66	0.93	0.19	0.03–1.18
Verbal assault—yes	−0.14	0.55	0.86	0.29–2.59	0.50	0.37	0.60	0.28–1.26	0.79	0.49	2.20	0.83–5.85
Psychological assault—yes	1.14	1.37	3.13	0.21–46.08	0.31	0.79	1.36	0.28–6.44	0.42	1.03	1.52	0.20–11.51
Assault by patients—yes	0.29	0.45	1.34	0.54–3.28	0.54	0.31	1.72	0.92–3.19	0.78 ^∗^	0.38	2.19	1.04–4.64
Assault by patients’ relatives—yes	−0.71	0.49	0.49	0.18–1.28	0.33	0.31	1.40	0.75–2.60	0.32	0.37	1.38	0.65–2.89
Assault by colleagues—yes	0.41	0.46	1.50	0.61–3.72	0.49	0.29	1.64	0.92–2.94	1.00 ^∗∗^	0.36	2.71	1.32–5.57
Care of patients with suicide ideation—yes	0.06	0.30	1.06	0.58–1.93	0.35 ^∗^	0.19	1.42	1.01–2.08	−0.21	0.27	0.80	0.47–1.39
Work‐related distress	0.001	0.005	1.001	0.99–1.01	0.01 ^∗^	0.03	1.01	1.009–1.02	0.02 ^∗∗∗^	0.005	1.02	1.01–1.03

^∗^ *p* ≤ 0.05.

^∗∗^ *p* ≤ 0.01.

^∗∗∗^ *p* ≤ 0.001.

### 3.5. COVID‐19 as a Confounder

In Mexico, the timeline from 2019 to the end of 2021 spans the prepandemic period and the phase of sustained community transmission following the declaration of Phase 3 on April 21, 2020, by the Ministry of Health in Mexico. A sensitivity analysis was conducted comparing the prevalence of depression, anxiety, and both depression and anxiety among residents assessed during the prepandemic period (prior to March 24, 2020) with those evaluated during the community transmission phase (after March 24, 2020), which was characterized by widespread transmission and coincided with the national vaccination rollout in 2021 [18]. This grouping allowed for the comparison of residents in clearly differentiated epidemiological contexts.

The sensitivity analysis based on the COVID‐19 stages in Mexico showed no significant differences in the distribution of mental health outcomes between the prepandemic and the transmission phase groups (*p*  > 0.05). In the prepandemic period, 71.7% (*n* = 428) of residents were classified as controls, 7.4% (*n* = 44) presented depression, 14.1% (*n* = 84) anxiety, and 6.9% (*n* = 41) comorbidity. Similarly, during the transmission phase, 77.3% (*n* = 472) were controls, 3.3% (*n* = 20) had depression, 12.1% (*n* = 74) had anxiety, and 7.4% (*n* = 45) comorbidity. These findings indicate that the prevalence of depression, anxiety, and their comorbidity remained stable across the different epidemiological periods, supporting the robustness of the main results regardless of the pandemic stage at the time of assessment.

## 4. Discussion

This investigation provides valuable insights into the mental health challenges faced by anesthesiology residents, highlighting factors that specifically contribute to the presence of depression, anxiety, or both disorders in comorbidity among this vulnerable population.

The response rate for our study was 88.6% of the 1363 anesthesiology residents in Mexico at the time of the survey. Notably, 25.5% of the residents reported experiencing depression, anxiety, or both at the time of the survey, a prevalence significantly higher than that reported among the general population over 18 years old in Mexico: 6.4% for depression and 3.85% for anxiety [19]. However, compared to other studies involving anesthesiology residents and specialists, we observed a slightly lower prevalence. It is important to note that most existing research on the mental health of anesthesiologists has primarily focused on the impact of the COVID‐19 pandemic [11, 12, 20–22]. In contrast, our study was designed to provide a comprehensive evaluation of the overall experience of anesthesiology residents throughout their entire residency period rather than concentrating solely on pandemic‐related factors. This broader approach enables a more holistic understanding of their mental well‐being beyond the context of the pandemic. Although the sensitivity analysis showed no differences between residents assessed before the COVID‐19 pandemic and those evaluated during the transmission phase, the potential impact of the pandemic on the mental health of residents in Mexico and worldwide should not be disregarded as its effects may have emerged at a later stage in their professional lives [23].

Despite the growing interest in physicians’ mental health, there is still a vast lack of knowledge regarding some of the fundamental aspects of physician well‐being, including their mental health and the challenges they face. While there are common factors in the medical practice that can contribute to the development of depression and/or anxiety, such as workload and long working shifts [6], each specialty may also present unique challenges that have a greater impact on residents’ mental health. The risk factor for depression in anesthesiology residents included not having a partner, while protective factors were related to sleep, specifically the number of hours and the perception of restful sleep, which also served as a protective factor for residents suffering from comorbid depression and anxiety.

In some cases, being single can exacerbate the risk of mental health problems for anesthesiology residents. Human beings have an intrinsic need for connection and belonging, both physically and emotionally. During residency, the overwhelming demands on time, energy, and professional identity can quickly erode personal well‐being and diminish availability for meaningful social connection. Unrealistic expectations of the residency and a lack of preparation often strain personal relationships [24]. When residents prioritize their professional identity over their personal lives, they may experience what’s known as “surface acting”—the act of presenting a professional facade while concealing true emotions [25]. This phenomenon can be particularly detrimental when residents lack close relationships or a partner, who might otherwise provide emotional support and help mitigate the emotional toll of the profession. Physical isolation inherent to anesthesia practice, combined with training demands that limit social engagement, create conditions that are particularly conducive to loneliness and subsequent mental health burdens. Personal relationships and a sense of belonging act as protective factors against stress. A sense of “thwarted belonging” arises when the human needs for connection, social companionship, and understanding are not fulfilled. Additionally, surface acting is more likely to generate negative emotions, leading to a narrow‐minded outlook, critical self‐judgment, and avoidance [4]; all of these are linked to increased vulnerability to mental health challenges, such as depression and anxiety [26].

Sleep is an essential biological process, and inadequate sleep, as well as untreated sleep disorders, can have significant negative consequences for health, well‐being, and public safety. While some studies indicate that fatigue or sleeping less than four consecutive hours during a 24‐h shift does not substantially affect medical reasoning [27], longer shift durations have been associated with an increased risk of errors, daytime sleepiness, diminished academic performance, decreased ability to work effectively in team settings, and adverse effects on healthcare providers’ health and well‐being [28]. Furthermore, poor sleep quality is a primary factor contributing to the heightened risk of secondary mental health disorders, including anxiety and depression [29]. Although the maximum working hours per day were consistent across the groups in this study, anesthesiology residents with depression or comorbid depression and anxiety reported a reduced frequency of restful sleep. This suggests that fatigue may impair both academic and clinical performance, particularly in the management of crisis situations. These obstacles can induce frustration and emotional fatigue, fostering depersonalization as a coping strategy, thereby diminishing personal fulfillment while engendering a feeling of inability to meet the demands of residency [27, 29], which increases the risk of depression among these individuals.

Distress is generally defined as unpleasant subjective stress responses that reflect psychobiological, cognitive, and social influences, but it should be understood as the outcome of dynamic self‐regulation as the person confronts external threats and pressures [30]. Apart from psychological aspects, when the organism perceives a threat, the primary manifestation is anxiety [31]. This aligns with our results, where work‐related distress increased the likelihood of experiencing anxiety or comorbid anxiety and depression among anesthesiology residents. Even though the odds ratios for this variable are low and the findings may not be clinically significant in terms of increased distress, it is important to consider the role it plays in terms of the residents’ mental health.

Anesthesiologists face high‐stress working conditions as anesthesia is a critical component of surgical procedures [7]. They work unpredictable hours and often perform under time pressure in situations that are potentially life‐threatening for patients [32]. For residents, the fear of failure and negative evaluation by instructors, peers, or their fellow surgeons can further exacerbate distress [31]. For those nearing the end of their training, distress may be linked to increasingly unsupervised work [33] as they take on a more active role in decision‐making regarding patient management. This transition to greater autonomy can generate additional distress due to the responsibility and the high‐stakes decisions that they must make in critical situations.

It is uncommon for anesthesiology residents to work with patients who suffer from suicidal ideation. However, it is important to consider that many patients may have pre‐existing psychiatric conditions, and the perioperative process—before, during, and after surgery—may exacerbate psychiatric symptoms, including suicidal ideation and aggressive behavior. The effective management of suicidal patients often requires collaboration with psychiatrists and other mental health professionals. This is crucial for ensuring psychiatric evaluation by the psychiatric team, and if a patient under anesthesiology care presents with a psychiatric condition, it is essential to have the support of liaison psychiatrists for the proper management of suicidal ideation [34–37]. Assaults from colleagues represented the factor that most increased the likelihood of comorbid depression and anxiety among anesthesiology residents. This phenomenon is particularly prevalent in medicine in an environment characterized by a hidden curriculum, where practices during training involve violent and intimidating scenarios [1] that probably relate to the immense responsibility that medical professionals bear for the health and lives of others. Anesthesiologists bear the pressure of ensuring patients’ safety during critical conditions [7], where any mistake could have fatal consequences.

Furthermore, this takes place in a medical culture where mistakes are not tolerated and behaviors, such as mistreatment and highly unrealistic expectations, have become normalized [38] as teaching methods. Medical training in Mexico occurs within a rigid hierarchical culture that normalizes the humiliation and mistreatment of students and residents as part of learning or a rite of passage. Doctors are stratified by training stage and specialty prestige, with surgical fields often valued more highly, while specialties such as anesthesiology are frequently perceived as less important [39–41]. While deeply embedded in the Mexican medical culture, teaching through humiliation is not unique to Mexico and is observed worldwide, with negative effects on learning, well‐being, and mental health. A meta‐analysis of 177 studies conducted across various countries revealed that 63.4% of medical residents reported experiencing harassment during their training [42]. These harmful experiences, especially when coupled with a lack of adequate support, can reach a tipping point, leading to emotional and mental health struggles, such as depression and anxiety [6]. Additionally, in Mexico, cultural values emphasizing respect for authority and endurance under adverse conditions, together with limited institutional support for mental health, may discourage help‐seeking behaviors and contribute to the underreporting of psychological distress, mental health problems, and workplace violence during residency training.

Monitoring the mental health of residents is essential, particularly for those with comorbid depression and anxiety, which represents 7.1% of the residents in our sample, who are highly vulnerable to severe symptoms, with the most severe risk profile for suicidal behavior, functional impairment, and burnout. In this study, they reported higher work‐related distress, greater exposure to assaults, and less restful sleep, highlighting the need for targeted interventions addressing the specific challenges faced by anesthesiology residents.

### 4.1. Strengths and Limitations

A major strength is the recruitment of a large sample of anesthesiology residents from Mexico. When assessing depression and anxiety, physicians often under‐ or over‐report symptoms [43] due to the lack of objective evaluations by mental health professionals and reliance on self‐report tools. Although the ICD‐11 PHC screening test is a validated and practical tool for identifying depressive and anxious symptoms in professional settings, we acknowledge a potential false‐positive rate of 11%–16%. This margin of error highlights that screening results should not replace formal clinical evaluations. These findings underscore the urgent need for structured mental health support and definitive diagnostic assessments specifically tailored for anesthesiology residents.

Part of the sample was obtained via email forwarding (snowball sampling), which may have introduced selection bias, limited control over the sampling frame, and prevented the calculation of an accurate response rate, thereby potentially reducing the representativeness and generalizability of the findings. Despite the limitations inherent to this recruitment method, the present study included anesthesiology residents from 31 of the 32 federal states in Mexico, providing broad geographic representation. Although there was no absolute verification that all respondents were anesthesiology residents, the survey was distributed through specialty‐specific professional and academic networks. Given the high cohesion and interconnectedness typically observed among resident communities, participation by individuals outside the target population is considered unlikely, although it cannot be completely ruled out.

Another limitation concerns the assessment of key variables such as sleep quality and work‐related stress using single‐item measures. Although this approach reduced the respondent burden and facilitated data collection, it may have limited the reliability, sensitivity, and construct validity of these measures, preventing a more nuanced evaluation of these multidimensional constructs. Future studies would benefit from the use of validated multi‐item instruments that allow for a more comprehensive and robust assessment. Although the data are based solely on self‐reports, the anonymous online survey used in our study helped reduce stigma and encouraged honest responses. However, the use of self‐report instruments is also an important limitation to be considered. Responses may be influenced by social desirability and individual response styles, introducing reporting bias. Additionally, the retrospective nature of some questions may lead to recall bias, potentially affecting the accuracy of the information provided. Given the cross‐sectional design, it is important to emphasize that the results only reveal associations and not causality. The temporal sequence between exposure and outcome cannot be established, which precludes determining whether the identified factors preceded or resulted from symptoms of depression and anxiety. Additionally, cross‐sectional studies are particularly vulnerable to reverse causality and residual confounding, as unmeasured or inadequately controlled variables may partially explain the observed associations. This design also limits the ability to assess changes over time, trajectories of symptom development, or the persistence and fluctuation of mental health conditions. The finding for single marital status in the group of residents with depression should be interpreted with caution due to the sample size and because it barely reached statistical significance. The multinomial logistic regression model showed a Nagelkerke *R* ^2^ of 13.3%, indicating a modest explanatory power of marital status, sleep, assault, and distress. This suggests that additional unmeasured factors—such as personality traits, substance use, mental health history, and work environment perceptions—may have a significant contribution to the outcomes and should be further investigated. This limitation should be considered when interpreting the findings and highlights that further research is needed to explore these associations and identify key risk factors for depression and anxiety. Regarding external validity, the findings of this study may not generalize to other surgical or nonprocedural specialties. Additionally, cultural and structural differences in medical training limit the extrapolation of these results to other countries.

## 5. Conclusions

Medical specialty residents represent key pillars of the healthcare system, whose performance relies heavily on their knowledge, skills, and motivation. The mental health of anesthesiology residents is crucial to their effectiveness. The challenges they face—long hours, highly pressurized situations, and emotional strain—can significantly affect both their well‐being and clinical performance. The quality of medical education is shaped not only by academic instruction but also by social dynamics with patients, families, supervisors, and peers. A healthier, more supportive medical environment, including targeted interventions such as protecting residents’ sleep, implementing strict caps on shift duration, and fostering psychological safety, would improve anesthesiology residents’ mental well‐being, leading to greater job satisfaction, fewer mental health issues, and enhanced patient care.

Addressing mental health among anesthesiology residents requires systemic changes. This is a highly complex profession defined by the continuous need for management of unpredictable critical situations. To maintain clinical performance, these challenges must be faced with an equivalent increase in understanding, acceptance, guidance, and care for these professionals, as it is clear that their health will be affected. Institutions must prioritize mental health through regular screenings, supportive peer networks, and a culture where residents feel comfortable discussing their challenges. This would reduce stigma and encourage residents to seek help while supporting their mental health. Improving residents’ mental health would enhance their clinical performance, ultimately improving patient care and contributing to a more effective healthcare system. Focusing on mental health will help create a more resilient, motivated, and capable workforce, ready to meet the demands of modern medicine.

For anesthesiology residency programs, the challenge is clear: to balance the intensive training requirements of this critical specialty with sustainable practices that protect resident well‐being and, by extension, ensure optimal patient care. Continued research, particularly longitudinal studies tracking the long‐term impact of wellness interventions, will be valuable in refining approaches to this complex issue.

## Author Contributions

**Diana Guízar-Sánchez**: conceptualization, methodology, validation, writing – original draft. **Ana Fresán**: conceptualization, methodology, formal analysis, supervision, writing – original draft. **Ricardo-Arturo Saracco-Alvarez**: investigation, data curation, writing – original draft. **María Yoldi-Negrete**: conceptualization, methodology, writing – original draft. **Rebeca Robles-García**: methodology, validation, writing – original draft. **Carlos-Alfonso Tovilla-Zárate**: validation, investigation, visualization, writing – original draft.

## Funding

No funding was received for this research.

## Ethics Statement

All study procedures were approved by the Ethics and Research Committees of the Ramón de la Fuente Muñiz National Institute of Psychiatry, Secretariat of Health, Mexico (Approval Numbers CEI/C/018/2019 and IC19119.0). Participants took part in this study on a voluntary basis, were not paid for their participation, and could withdraw their consent by not completing the online survey. Anonymity was ensured and participants had the option to withdraw at any time by exiting the survey.

## Conflicts of Interest

The authors declare no conflicts of interest.

## Data Availability

The data that support the findings of this study are available from the corresponding author upon reasonable request.
